# Genotype-Specific Minimal Residual Disease Interpretation Improves Stratification in Pediatric Acute Lymphoblastic Leukemia

**DOI:** 10.1200/JCO.2017.74.0449

**Published:** 2017-11-13

**Authors:** David O’Connor, Amir Enshaei, Jack Bartram, Jeremy Hancock, Christine J. Harrison, Rachael Hough, Sujith Samarasinghe, Claire Schwab, Ajay Vora, Rachel Wade, John Moppett, Anthony V. Moorman, Nick Goulden

**Affiliations:** David O’Connor, Jack Bartram, Sujith Samarasinghe, Ajay Vora, and Nick Goulden, Great Ormond Street Hospital; Rachael Hough, University College Hospital, London; Amir Enshaei, Christine J. Harrison, Claire Schwab, and Anthony V. Moorman, Northern Institute for Cancer Research, Newcastle University, Newcastle upon Tyne; Jeremy Hancock, North Bristol National Health Service Trust; John Moppett, Royal Hospital for Sick Children, Bristol; Ajay Vora, Sheffield Children’s Hospital, Sheffield; Rachel Wade, Medical Research Council, University of Oxford, Oxford, United Kingdom; and Nick Goulden, Trapehade, Monferran-Plavès, France.

## Abstract

**Purpose:**

Minimal residual disease (MRD) and genetic abnormalities are important risk factors for outcome in acute lymphoblastic leukemia. Current risk algorithms dichotomize MRD data and do not assimilate genetics when assigning MRD risk, which reduces predictive accuracy. The aim of our study was to exploit the full power of MRD by examining it as a continuous variable and to integrate it with genetics.

**Patients and Methods:**

We used a population-based cohort of 3,113 patients who were treated in UKALL2003, with a median follow-up of 7 years. MRD was evaluated by polymerase chain reaction analysis of *Ig/TCR* gene rearrangements, and patients were assigned to a genetic subtype on the basis of immunophenotype, cytogenetics, and fluorescence in situ hybridization. To examine response kinetics at the end of induction, we log-transformed the absolute MRD value and examined its distribution across subgroups.

**Results:**

MRD was log normally distributed at the end of induction. MRD distributions of patients with distinct genetic subtypes were different (*P* < .001). Patients with good-risk cytogenetics demonstrated the fastest disease clearance, whereas patients with high-risk genetics and T-cell acute lymphoblastic leukemia responded more slowly. The risk of relapse was correlated with MRD kinetics, and each log reduction in disease level reduced the risk by 20% (hazard ratio, 0.80; 95% CI, 0.77 to 0.83; *P* < .001). Although the risk of relapse was directly proportional to the MRD level within each genetic risk group, absolute relapse rate that was associated with a specific MRD value or category varied significantly by genetic subtype. Integration of genetic subtype–specific MRD values allowed more refined risk group stratification.

**Conclusion:**

A single threshold for assigning patients to an MRD risk group does not reflect the response kinetics of the different genetic subtypes. Future risk algorithms should integrate genetics with MRD to accurately identify patients with the lowest and highest risk of relapse.

## INTRODUCTION

The assessment of treatment response via the measurement of minimal residual disease (MRD) is now recognized as the most powerful prognostic factor in acute lymphoblastic leukemia (ALL).^1-4^ The integration of MRD monitoring into risk-adapted protocols has been used to successfully guide therapy intensification and reduction^2,5-7^; however, MRD alone is not sufficient to fully predict outcome. Somatic genetic abnormalities define fundamentally distinct biologic subgroups, and several are important prognostic and predictive biomarkers.

The extent to which the presence of specific genetic abnormalities influences the kinetics of disease clearance is not fully understood, and there is no consensus surrounding the best method for integrating genetic and MRD data to stratify patients. Although analysis of the BFM-2000 trial led to the conclusion that molecular response redefines all prognostic factors, only a handful of genetic abnormalities were considered and *ETV6-RUNX1* retained its significance in the multivariable model.^8^ In addition, studies by the Children’s Oncology Group (COG) and St Jude Children’s Research Hospital have noted significant associations between genetic abnormalities and MRD.^9,10^ Previous studies of specific genetic subgroups have also led to different conclusions. For example, the United Kingdom and COG study groups assign patients with iAMP21 (intrachromosomal amplification of chromosome 21) to high-risk (HR) regimens irrespective of MRD,^11,12^ whereas the BFM study group relies on MRD to assign risk in these patients.^13^ Other studies of low hypodiploidy and Philadelphia chromosome–like ALL have proposed that treatment response can refine risk for patients with these abnormalities, but most protocols still allocate these patients to HR therapy.^14,15^

A common feature of these previous studies is the use of categorical variables with which to study MRD. Dichotomization of continuous variables leads to the loss of statistical power equivalent to removing one third of data.^16^ To fully explore the discriminatory power of MRD and examine its interaction with genetics, we analyzed a large, well-annotated cohort of patients who were treated in a single trial using MRD as a continuous variable to study response at the end of induction (EOI).

## PATIENTS AND METHODS

**Fig A1.**
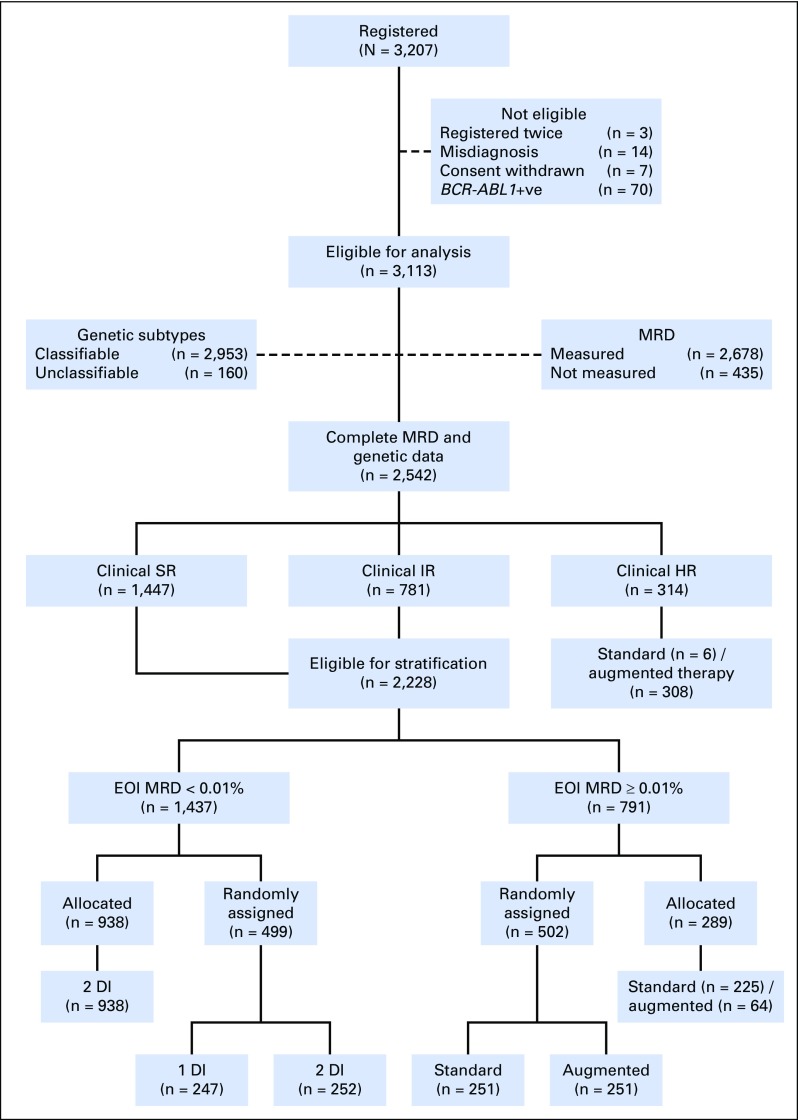
CONSORT diagram for UKALL2003 showing the number of patients who were available for analysis in this study and the random assignment and/or major treatment pathways. Patients who had > 25% of the bone marrow made up of blast cells at day 8 (National Cancer Institute [NCI] high risk) or 15 (NCI standard risk) were reclassified to the clinical high-risk group irrespective of their initial classification and were not eligible for minimal residual disease (MRD) stratification and random assignment. NCI standard-risk (SR) patients had to have an early response of < 25% marrow blasts at the day 15 assessment (reclassified as clinical SR) and NCI high-risk (HR) patients < 25% marrow blasts at day 8 (reclassified as clinical intermediate risk [IR]) to be eligible for random assignment if they were younger than 16 years of age. All patients ≥ 16 years of age were treated as clinical IR irrespective of day 8 or day 15 bone marrow response and were eligible for MRD stratification and random assignment. Morphologic remission status was assessed at day 29 of induction and complete remission was defined as a marrow blast count < 5%. Patients who were not in complete remission at day 29 of induction were not eligible for MRD stratification and random assignment. We stratified clinical SR and IR groups by bone marrow MRD at the end of induction (EOI; day 29 from the start of treatment). augmented therapy, transferred to regimen C; DI, delayed intensification; standard therapy, remaining on regimen A/B.

A total of 3,113 consecutive patients who were diagnosed with ALL by standard flow cytometric criteria and who were treated in the MRC UKALL2003 (2003 to 2011) trial were available for analysis (Appendix Fig A1, online only).^2,5^ The trial was approved by the Scottish Multi-Centre Research Ethics Committee, and written informed consent was obtained from parents and patients in accordance with the Declaration of Helsinki. Full details of the treatment protocol and results of the main trial questions have been reported.^2,5^

**Fig A2.**
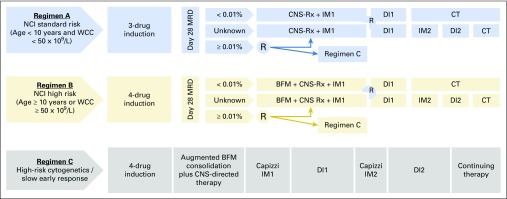
Schematic representation of the ALL2003 treatment protocol. Regimen A was composed of a three-drug induction—vincristine, dexamethasone, and asparaginase—followed by consolidation (daily mercaptopurine and weekly intrathecal methotrexate), CNS-directed therapy, interim maintenance (daily mercaptopurine, weekly methotrexate, monthly vincristine, and corticosteroid pulses), delayed intensification (asparaginase, vincristine, dexamethasone, and doxorubicin), and continuing therapy (oral mercaptopurine and methotrexate, monthly vincristine and corticosteroid pulses, and intrathecal methotrexate every 3 months). Regimen B patients also received daunorubicin during induction and Berlin Frankfurt Munster (BFM) consolidation (4 weeks of cyclophosphamide and cytarabine). Regimen C patients received an additional four doses of vincristine and two doses of pegylated asparaginase during BFM consolidation. Furthermore, regimen C patients received escalating doses of intravenous methotrexate without folinic acid rescue, and vincristine and pegylated asparaginase as interim maintenance (Capizzi maintainance). CT, continuing therapy; DI, delayed intensification; IM, interim maintenance; MRD, minimal residual disease; NCI, National Cancer Institute; WCC, white cell count.

Initially, National Cancer Institute (NCI) standard-risk (SR) patients (< 10 years and white cell count < 50 × 10^9^/L) were assigned to regimen A, whereas NCI HR patients (≥ 10 years and/or white cell count ≥ 50 × 10^9^/L) received regimen B (Appendix Fig A2, online only). Patients with HR cytogenetics and patients age less than 16 years with a slow early response were assigned to regimen C. Slow early response was defined as ≥ 25% blasts in the day 15 (NCI SR) or day 8 (NCI HR) marrow. MRD was evaluated by real-time quantitative polymerase chain reaction analysis of *Ig/TCR* gene rearrangements with a quantitative range of 0.01% as defined by the European MRD Study Group.^17^ Patients with undetectable MRD at EOI (day 29) and before interim maintenance were classified as MRD low risk, as were those who had detectable EOI MRD (< 0.01%), but undetectable MRD before the start of interim maintenance. MRD low-risk patients were eligible for treatment reduction random assignment. Patients with EOI MRD ≥ 0.01% were classified as MRD HR and were eligible for treatment intensification randomization.

**Fig A3.**
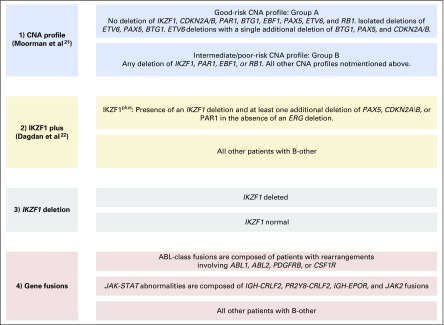
Four methods of subclassifying B-other acute lymphoblastic leukemia by genetics. For *CDKN2A/B*, deletion of either the *CDKN2A* or *CDKN2B* probes were sufficient for the locus to be classified as deleted. For *PAX5*, intragenic amplifications were coded with the deletions, as they are predicted to be functionally equivalent. A deletion in the PAR1 region of chromosome X or Y—del(X)(p22.33p22.33)/del(Y)(p11.32p11.32)—results in the loss of the *CSF2RA* and *IL3RA* probes, but the retention of the *CRLF2* probe. CNA, copy number alteration.

Cytogenetic and fluorescence in situ hybridization testing was performed, and data were curated as previously reported.^18^ Patients were classified into four mutually exclusive genetic groups: cytogenetic good risk (CYTO-GR): *ETV6-RUNX1*, high hyperdiploidy (51 to 65 chromosomes); cytogenetic HR (CYTO-HR): *KMT2A* (*MLL*) fusions, near haploidy, low hypodiploidy (< 40 chromosomes), iAMP21, and *TCF3-HLF*; cytogenetic intermediate risk (CYTO-IR): *TCF3-PBX1* and all other patient-cases with abnormal or normal cytogenetics (B other); and patients with T-ALL.^18^ Copy number alterations (CNAs) affecting *IKZF1*, *CDKN2A/B*, *PAX5*, *EBF1*, *ETV6*, *BTG1*, *RB1,* *PAR1*, and *ERG* were assessed by multiplex ligation-dependent probe amplification using the SALSA P335/P327 kits (MRC Holland, Amsterdam, the Netherlands) and SNP6.0 array (Affymetrix, Santa Clara, CA) as previously described.^19,20^ B-other patients were subclassified into previously defined subgroups on the basis of CNA^21,22^ (Appendix Fig A3, online only).

Survival analysis considered three end points: event-free survival (EFS), defined as time to relapse, second tumor, or death, with censoring at the date of last contact; relapse rate (RR), defined as the time to relapse for those who achieved a complete remission, with censoring at the date of death in remission or last contact; and overall survival (OS), defined as the time to death, with censoring at the date of last contact. Patients were observed to March 1, 2016, giving a median follow-up time of 7 years. Survival rates were calculated and compared by using Kaplan-Meier methods, log rank tests, and Cox proportional hazards regression models (univariable and multivariable analyses). To examine MRD as a continuous variable, we assigned patient-cases with undetectable MRD a value one log below the minimum detection level of 1 × 10^−5^ and assumed a maximum value of 0.99999. The absolute value of natural log of this transformed MRD value is referred to as τ(MRD). Normality was assessed by using the skewness and kurtosis, Shapiro-Wilk and Shapiro-Francia tests. Log normal distributions were compared by using a multiple-sample, multivariable test of means. As a result of the investigative nature of this analysis, all tests were conducted at the 1% significance level. Analyses were performed by using Intercooled STATA (version 14.01; STATA, College Station, TX; Computing Resource Center, Santa Monica, CA).

## RESULTS

### Examining MRD as a Continuous Variable

**Fig 1.**
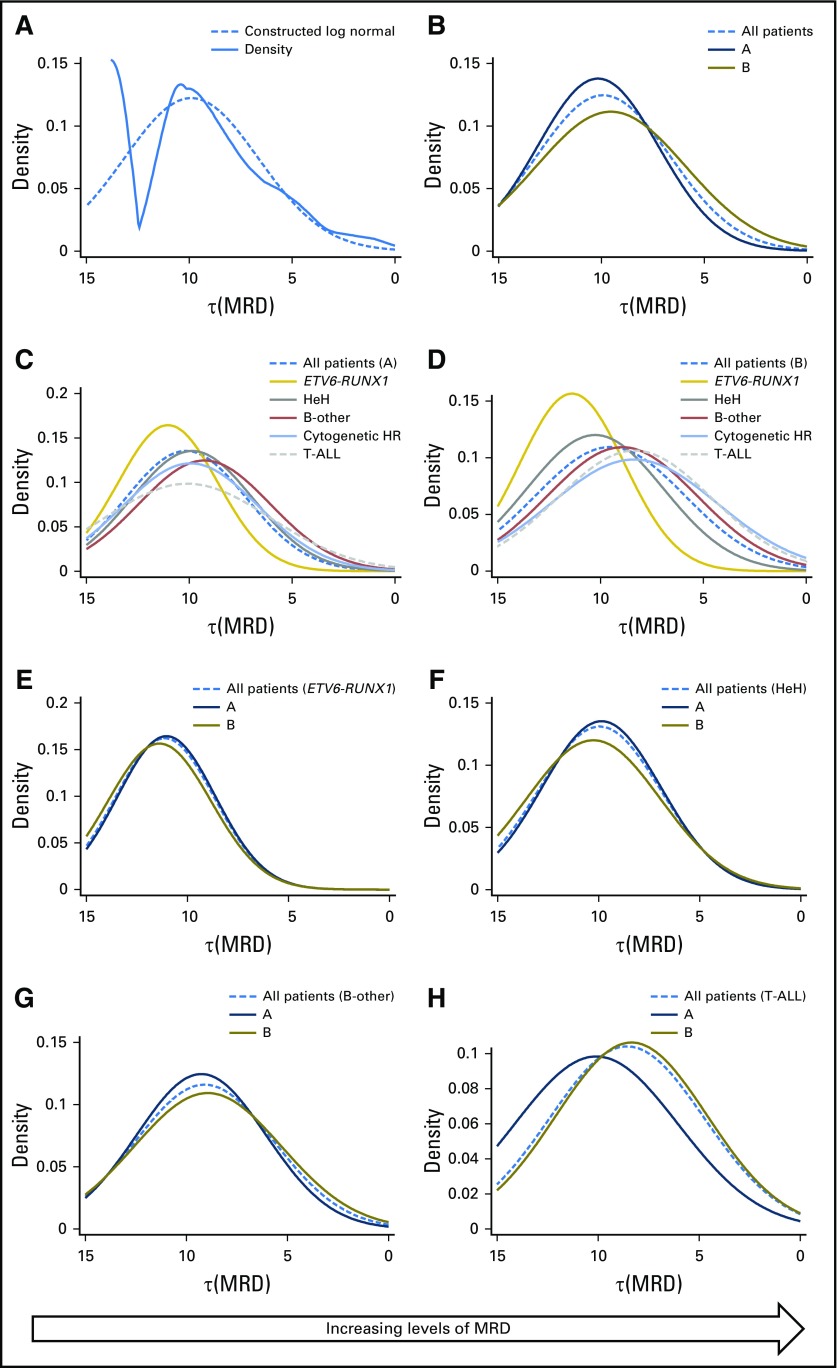
Distribution of the log transformed minimal residual disease (MRD) value, τ(MRD). (A) Raw (solid) and smoothed (dotted) density plots of τ(MRD) for 2,678 patients treated on UKALL2003. (B) Smoothed log normal distributed of τ(MRD) by induction therapy: regimen A (dark blue) and regimen B (brown). (C and D) Smoothed log normal distributions of τ(MRD) stratified by genetics among patients treated on (C) regimen A and (D) regimen B. (E-H) Smoothed log normal distributions of τ(MRD) stratified by induction treatments for patients with (E) *ETV6-RUNX1*, (F) high hyperdiploidy (HeH), (G) B-other ALL, and (H) T-cell acute lymphoblastic leukemia (T-ALL). HR, high risk.

MRD was measured at the EOI for 2,678 (86%) patients who were treated in UKALL2003. To allow the detailed examination of the kinetics of treatment response and to compare different patient subgroups, we transformed the absolute MRD value to produce a continuous MRD variable. This log variable, τ(MRD), ranged from 0 (highest MRD value) to 15 (undetectable MRD; Fig 1A). Tests for normality and Q-Q plots indicated that τ(MRD) followed a truncated normal distribution. Thus, the first peak, comprising 744 (27.7%) patients who had undetectable MRD, represented the detection limit of the assay (1 × 10^−5^), rather than a biologic phenomenon. Hence, we hypothesized that τ(MRD) was normally distributed.

### Response Kinetics by NCI Risk Group and Genetics

Figure 1 and Appendix Table A1 (online only) detail the distribution of τ(MRD) by NCI risk and genetic groups. Patients who were classified as NCI SR received a three-drug induction (regimen A), whereas NCI HR patients received a four-drug induction (regimen B). Despite receiving more intensive induction, NCI HR patients, on average, had a slower response (*P* < .001); however, there was significant variation in response kinetics by genetic subtype, both in the overall cohort and when stratified by treatment.

Among the major subgroups, *ETV6-RUNX1* patients had the fastest disease clearance, with 36% (245 of 675) having undetectable MRD, whereas CYTO-HR/T-ALL patients recorded the slowest disease clearance (Figs 1C and 1D and Appendix Table A1). MRD was log-normally distributed within each genetic subtype (*P* > .1), with the exception of *ETV6-RUNX1* (*P* = .01). There was no difference in response kinetics for *ETV6-RUNX1*, high hyperdiploidy, or CYTO-IR patients according to induction treatment. Patients with T-ALL who were treated with regimen A had a significantly better response compared with those who were treated with regimen B.

**Fig 2.**
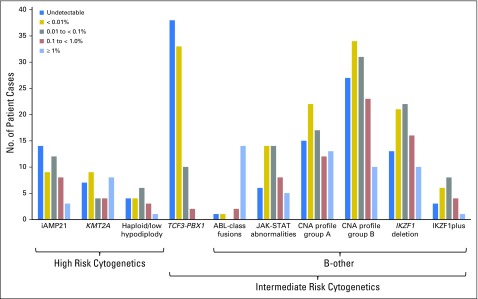
Distribution of selected genetic abnormalities by minimal residual disease category. B-other subcategories are not mutually exclusive and are defined in Appendix Fig A3. CNA, copy number alteration; iAMP21, intrachromosomal amplification of chromosome 21.

Several abnormalities, which were too infrequent to allow individual examination of τ(MRD), comprised the CYTO-HR group; therefore, we examined MRD distribution by category (Fig 2 and Appendix Table A2, online only). MRD was distributed normally for haploid, low hypodiploid, and iAMP21 patients, whereas among *KMT2A* patient-cases, MRD was more evenly spread and included a high proportion of refractory patients (19%). The CYTO-IR group was also heterogeneous, composed of *TCF3-PBX1* (10%) and B-other ALL (90%). Patients with *TCF3-PBX1* exhibited fast disease clearance with 38 (46%) of 83 patients having undetectable MRD. In contrast, the log normal MRD distribution for B-other patients was shifted to the right, which indicated slower disease clearance (Figs 1C and 1D).

To further investigate the B-other subgroup, we screened a representative subset of patients (n = 221) for CNA (Fig 2 and Appendix Fig A3).^21^ MRD of patients with a group A/B CNA profile, *IKZF1* deletion, and IKZF1^plus^ profile^22^ was log-normally distributed (*P* > .6). These B-other abnormalities are not mutually exclusive, and it is interesting to note that four of 13 patients with group A CNA profile, which is associated with a good outcome, and MRD ≥ 1% had an ABL-class fusion. MRD was also log-normally distributed among patients with activation of the JAK-STAT pathway compared with ABL-class fusion patients where 14 of 18 patients had MRD ≥ 1%.

### Integrating MRD and Genetics to Define New Clinically Relevant Subgroups

**Fig 3.**
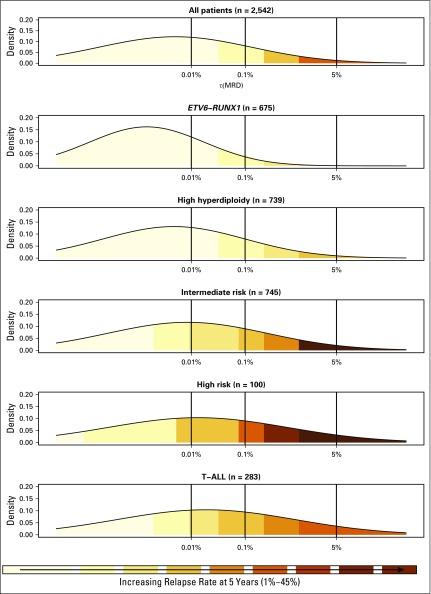
Relationship between minimal residual disease (MRD) at relapse risk. Each panel shows a smoothed density distribution of MRD for patient-cases in a particular genetic subtype. Shading corresponds to the risk of relapse for patients with that particular MRD level. The dotted line indicates the τ(MRD) value that corresponds to specific MRD values. T-ALL, T-cell acute lymphoblastic leukemia.

The construction of a normally distributed log-transformed MRD variable, τ(MRD), allowed outcome to be measured in relation to the log reduction in the leukemic cell population. Univariable Cox proportional hazards regression models for EFS, RR, and OS demonstrated that each log reduction in MRD equated to an approximate 20% decrease in the risk of an adverse event: EFS, 0.81 (95% CI, 0.78 to 0.83); RR, 0.80 (95% CI, 0.77 to 0.83); and OS, 0.77 (95% CI, 0.74 to 0.80). This effect was observed consistently across treatment, random assignment, and genetic subgroups, with the exception of one/two delayed intensifications (Table 1). Although the risk of relapse was directly proportional to the MRD level within each genetic risk group, the absolute risk of relapse that was associated with a specific MRD level varied by genetic subtype (Fig 3). To further illustrate this relationship and to aid stratification, we calculated 5-year EFS, RR, and OS rates for multiple MRD categories across genetic subtypes (Table 2). Survival varied significantly for a given MRD category. Patients with MRD levels at either end of the spectrum had similar outcomes, regardless of genetic subgroup, whereas the outcome that was associated with moderate MRD levels was genetic subtype dependent. Additional evidence that both MRD and genetics impact prognosis was evident when we examined some of the specific genetic abnormalities that underpin these broad cytogenetic risk groups (Appendix Table A2). Use of genetic-specific MRD thresholds to define risk groups enabled the creation of subsets with a more uniform outcome. In Table 2, we define exemplar risk groups by grouping together MRD-genetic subsets that have a low RR (< 7%) and a correspondingly high OS (> 94%) into a large SR group that accounts for two thirds of patients. The remaining patients were split into intermediate-risk and high-risk groups primarily on the basis of RR (< 20% or > 20%); however, all CYTO-HR patients were classified into the HR group because these outcomes were achieved by receiving HR treatment (regimen C).

**Table 1.**
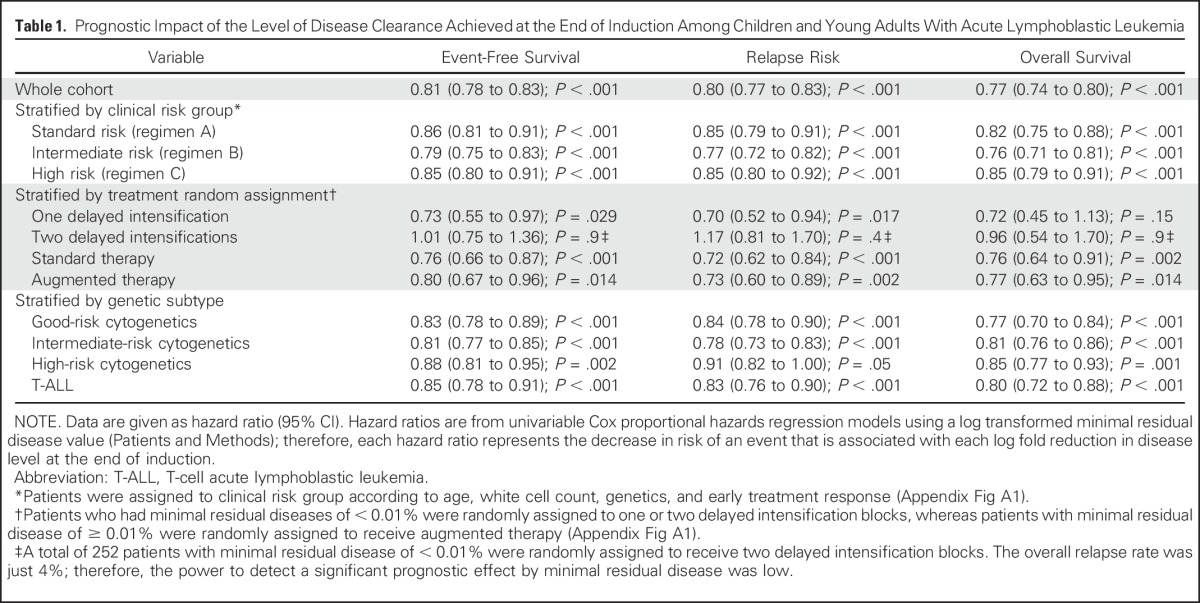
Prognostic Impact of the Level of Disease Clearance Achieved at the End of Induction Among Children and Young Adults With Acute Lymphoblastic Leukemia

**Table 2.**
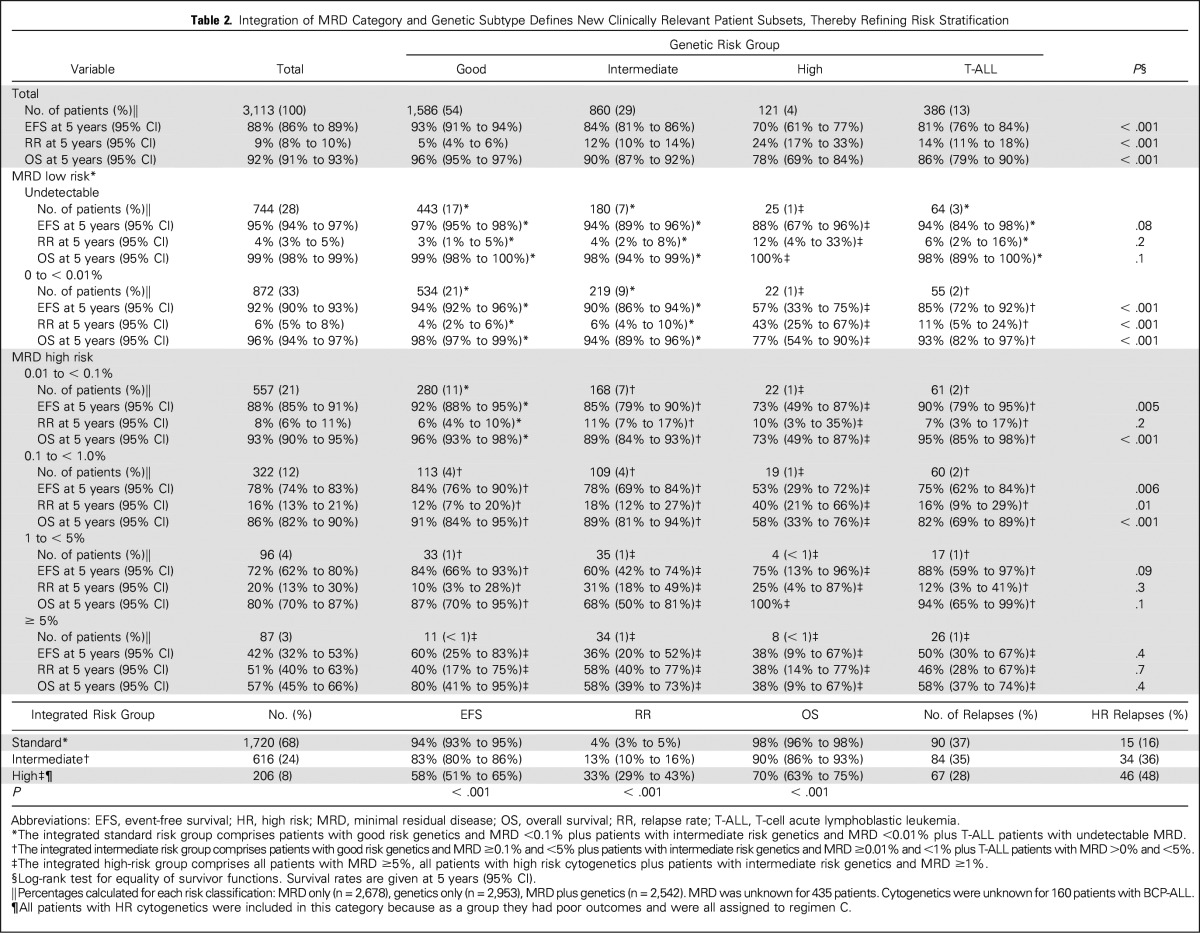
Integration of MRD Category and Genetic Subtype Defines New Clinically Relevant Patient Subsets, Thereby Refining Risk Stratification

### Correlation of MRD and Type of Relapse

**Fig 4.**
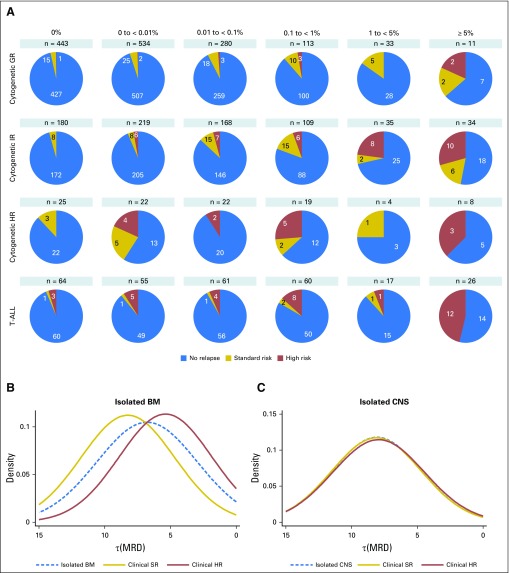
Diagram that shows the relationship between the end of induction (EOI) minimal residual disease (MRD) and the clinical risk group at relapse. High-risk (HR) relapses are composed of all early relapses (< 18 months from diagnosis), all T-cell acute lymphoblastic leukemia (T-ALL) bone marrow (BM) relapses and, in patients with BCP-ALL, early isolated marrow relapses with all other relapses classified as standard risk. (A) Pie charts illustrating the varying proportion of standard-risk (SR) and HR relapse patients by the level of MRD at EOI and genetic subtype. (B) Smoothed log-normal density plots for patients who experienced an isolated marrow relapse according to clinical risk group at relapse (*P* < .001). (C) Smoothed log normal density plots for patients who experienced an isolated CNS relapse according to clinical risk group at relapse (*P* = .95). GR, good risk; IR, intermediate risk.

At relapse, patients are classified as SR or HR on the basis of the time and site of relapse, and the majority of SR patients achieve a lasting second remission.^23,24^ The distribution of SR and HR relapses varied by genetic subtype and MRD (Fig 4A). The proportion of patients who experienced relapse that would be classified as clinical HR^23^ was strongly associated with genetic subtype: CYTO-GR, 20%; CYTO-IR, 38%; and CYTO-HR/T-ALL, 77% (*P* < .001). This association was observed across all MRD categories (Appendix Table A3, online only). By integrating MRD and genetics to define risk groups, it is possible to define a small HR group (8% patients) that captures 48% of HR relapses (Table 2). MRD assessment of marrow was not predictive of isolated CNS relapse. In keeping with this concept, we observed a significant difference in the MRD distributions for isolated marrow relapses by clinical risk group (Fig 4B), but not among patients who suffered an isolated CNS relapse (Fig 4C).

## DISCUSSION

Risk stratification is a key component of precision medicine, requiring the accurate measurement and integration of several prognostic factors to ensure appropriate treatment allocation. Whereas MRD and genetics have been shown to be the most important prognostic factors in ALL,^1-4,18^ they have largely been examined independently. We have reported, in unprecedented detail, the relationship between absolute MRD values and genetic abnormalities. These findings confirm and extend the observations by Pui et al,^9^ although analysis of independent data sets is required. Once validated, the concept of truly integrating MRD and genetics via subtype-specific MRD thresholds, as demonstrated by the integrated risk groups in Table 2, will improve risk algorithms that are used to allocate treatment.

To our knowledge, this study is the first to present MRD as a continuous variable and reveals the log normal distribution of MRD at EOI as predicted by the log cell kill effect of chemotherapy observed in mice and in vitro experiments.^25-27^ Whereas this distribution was maintained across different treatments and genetic subtypes, the kinetics of leukemic cell clearance differed. Perhaps unsurprisingly, mean MRD value was higher in NCI HR patients; however, this group received a four-drug induction that may have been expected to induce MRD levels comparable to NCI SR patients who received a three-drug induction. This suggests that a more intensive induction does not fully compensate for the inherent risk that is associated with NCI HR disease. An alternative explanation is that the addition of anthracycline simply does not add any efficacy to a three-drug dexamethasone-based induction. This is supported by the fact that the rate of true MRD negativity in our cohort (27.8%) was almost identical to that of the DCOG10 trial (28.8%), despite the universal use of a four-drug induction.^6^

MRD distributions differed to an even greater extent by genetic subtype, which indicated that the underlying disease biology is the key driver of treatment response and may have accounted for much of the difference between the NCI SR and HR groups. *ETV6-RUNX1* patients demonstrated a particularly rapid response to treatment that was consistent with their good prognosis. Unexpectedly, *TCF3-PBX1* patients, an intermediate-risk abnormality, demonstrated an even faster MRD clearance, with 43% achieving MRD negativity (Fig 2) compared with 36% of *ETV6-RUNX1* patients. These observations correlate with the recent report from the TCCSG L92-13 trial^28^ that showed that *ETV6-RUNX1* and *TCF3-PBX1* patients had an excellent outcome despite receiving less maintenance therapy. Thus, a greater understanding of disease kinetics could help tailor treatments to different subtypes.

Examining MRD as a continuous variable emphasizes that the relationship between MRD level and outcome is a continuum and that using a single cutoff value to stratify patients is an oversimplification. Moreover, integrating genetics aids the interpretation of this relationship and indicates that MRD alone is not sufficient to accurately stratify patients. Patients with MRD values at the extremes of the scale are the exception. Patients with high MRD (≥ 5%) have an extremely poor outcome (EFS of 42% at 5 years), irrespective of genetics, and should be considered for treatment intensification or novel therapies.^29^ Similarly, patients who achieve a true negative MRD response have an excellent outcome (EFS of 95% at 5 years) and may be suitable for treatment reduction in an effort to reduce toxicity. Of importance, even if patients with undetectable MRD do experience relapse, their disease is usually salvageable (Fig 4), providing additional reassurance that treatment reduction is an appropriate strategy. The log-normal distribution of MRD implies that some patients reduce their disease levels from the diagnostic burden of approximately 10^12^ leukemic cells^30^ by 6 to 7 logs after 4 weeks of therapy. Thus, there may be subset of patients whose disease can be eradicated by weeks, rather than years, of therapy. The development and application of ultrasensitive MRD methodologies will be required to test this hypothesis and identify such patients. Interpreting data for CYTO-HR patients with undetectable MRD is difficult because of modest numbers, genetic heterogeneity, and HR therapy. Of interest, all relapsed patients in this group had iAMP21 (Appendix Table A2). This finding supports the United Kingdom^11^ and COG^12^ conclusion that all patients with iAMP21 require HR treatment as opposed to the BFM finding that MRD alone identifies HR patients with iAMP21.^13^

Stratification of patients with MRD > 0%, but < 5%, is more complex but is informed by integrating genetics. To effectively stratify patients, it is imperative to use different MRD values within each group. For example, GR-CYTO patients with MRD of 0.01% to 0.1% had an excellent outcome, despite being classified as MRD HR and considered for treatment intensification. To assess the potential impact of therapy intensification, we examined the relapse rate among CYTO-GR patients who were randomly assigned to receive standard or augmented therapy (n = 157). Relapse rate remained low for all patients: 4.2% (1.4 to 12.4) versus 2.5% (0.6 to 9.8; *P* = .4). In our current trial, UKALL2011, we have now raised the threshold at which we classify CYT0-GR patients as being MRD risk. We anticipate that this intervention will reduce toxicity without comprising outcome. At the other end of the spectrum, CYTO-IR/MRD ≥ 1% and CYTO-HR/MRD > 0% patients have poor outcomes and could be considered together with patients with MRD ≥ 5% in an HR group. This strategy effectively delineates those patients with low risk and HR disease, leaving approximately one third of patients with intermediate outcomes who mainly have CYTO-IR or T-ALL.

The prognostic impact of MRD within T-ALL was intriguing. Patients with 0% or ≥ 5% MRD had excellent or poor outcomes, respectively, but otherwise MRD seemed to have little impact. Patients with T-ALL were significantly less likely to have a reportable MRD result: 73% versus 88% (*P* < .001). These observations could indicate that an MRD methodology that is reliant on *Ig/TCR* rearrangements is less useful in T-ALL; however, it should be noted that we have relatively few patients with T-ALL and were not able to examine the underlying genetic heterogeneity.^31^ CYTO-IR is also genetically heterogeneous. We demonstrated that many of the abnormalities that underpin B-other ALL will be useful in future algorithms to further refine genetic subtypes and, thus, new integrated risk groups. For example, patients with the group A CNA profile^21^ have an excellent outcome if MRD is < 1% and could be included in a low-risk group. Of interest, many of the patients with a group A CNA profile and MRD of > 1% harbored *EBF1-PDGFRB*.^32^

Risk stratification must be based on the efficacy and associated toxicity of the proposed treatments. Effective but toxic treatments—for example stem-cell transplantation—should be reserved for HR patients who are unlikely to be cured with conventional therapy; however, treatment intensification or novel agents may be considered appropriate for intermediate-risk patients. Currently, treatment protocols use a single MRD threshold to assign patients to risk groups, irrespective of the presence of genetic abnormalities; however, data generated by this study indicate that MRD must be interpreted within the context of genetics to maximize its effectiveness. Using different MRD cutoffs for different genetic subtypes allows more flexibility to define patient subgroups of the appropriate size and outcome. We propose that the future of stratification in ALL lies in the integration of MRD measurement with detailed genetic classification. We have already changed the MRD threshold for CYTO-GR patients in UKALL2011 and plan to use this strategy more fully when designing the risk stratification algorithm of our next trial.

## Figures and Tables

**Table A1.**
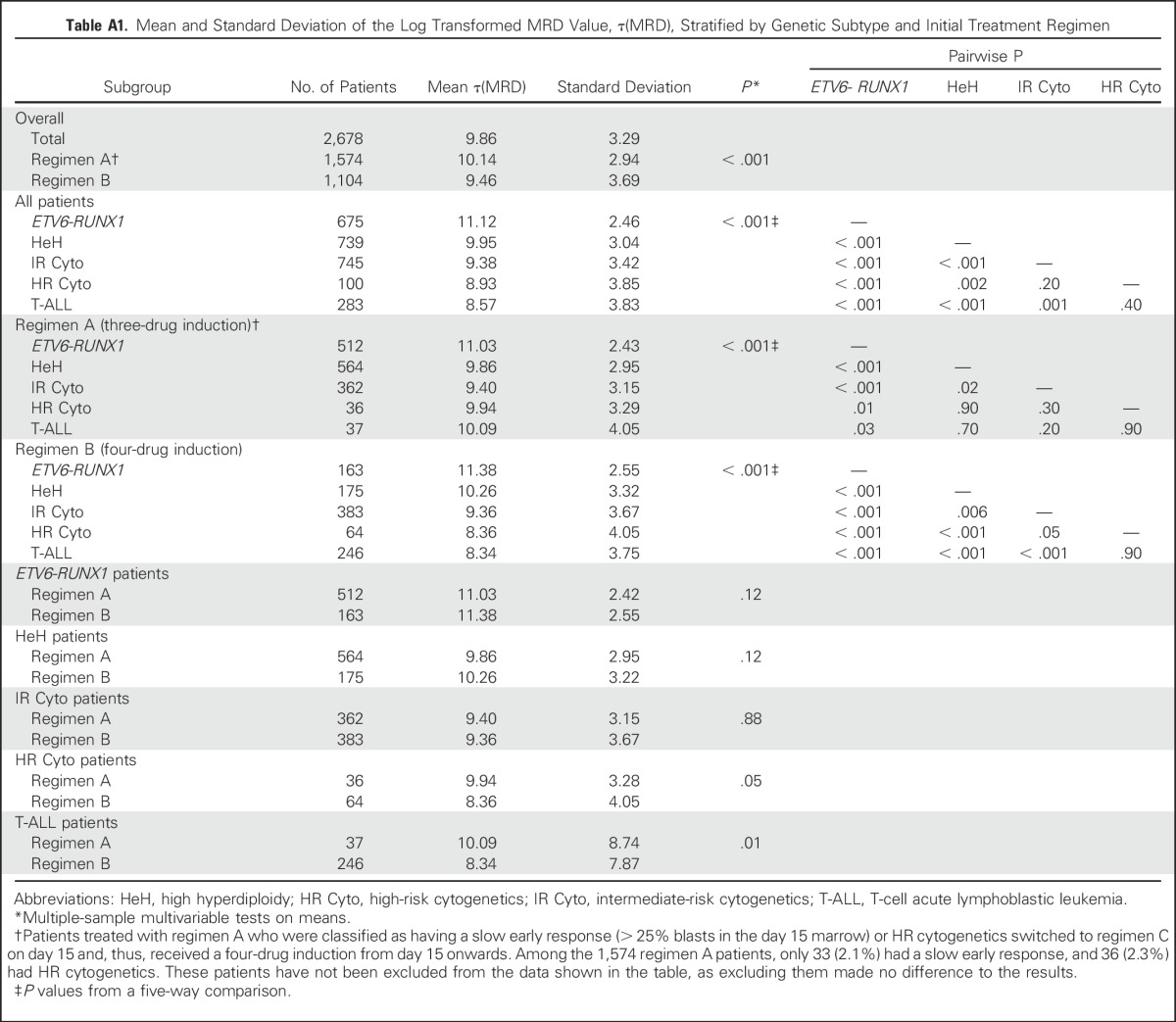
Mean and Standard Deviation of the Log Transformed MRD Value, τ(MRD), Stratified by Genetic Subtype and Initial Treatment Regimen

**Table A2.**
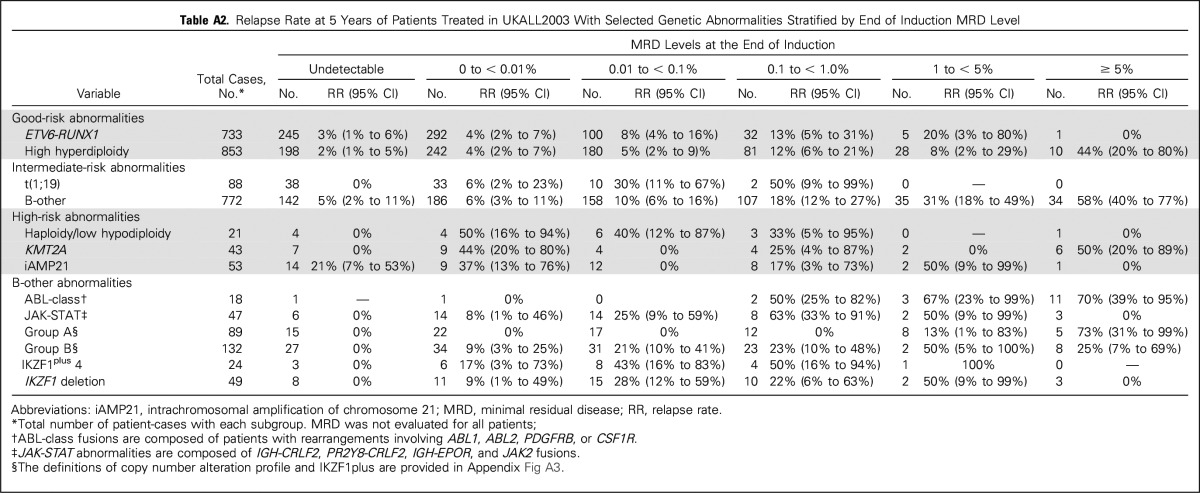
Relapse Rate at 5 Years of Patients Treated in UKALL2003 With Selected Genetic Abnormalities Stratified by End of Induction MRD Level

**Table A3.**
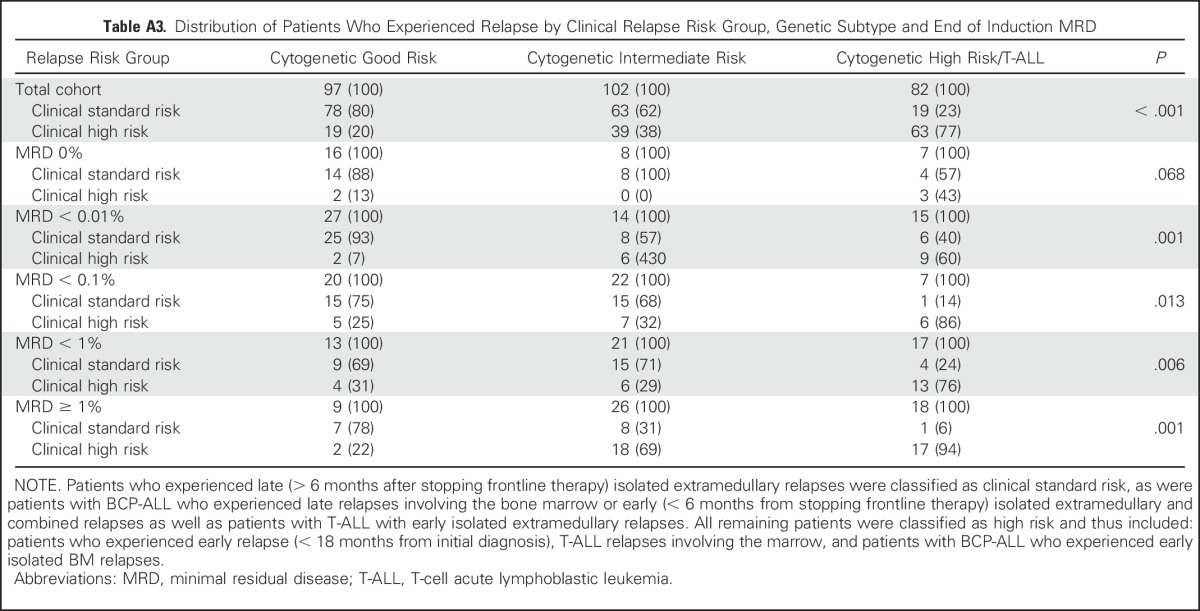
Distribution of Patients Who Experienced Relapse by Clinical Relapse Risk Group, Genetic Subtype and End of Induction MRD
